# Comparative effectiveness analysis of survival with first-line palbociclib or ribociclib plus AI in HR + /HER2- advanced breast cancer (CEPRA study): preliminary analysis of real-world data from Thailand

**DOI:** 10.1186/s12885-024-12765-x

**Published:** 2024-08-16

**Authors:** Thanate Dajsakdipon, Thiti Susiriwatananont, Concord Wongkraisri, Suthinee Ithimakin, Napa Parinyanitikul, Archara Supavavej, Arunee Dechaphunkul, Patrapim Sunpaweravong, Sunee Neesanun, Charuwan Akewanlop, Thitiya Dejthevaporn, TSCO Breast Oncology Group

**Affiliations:** 1 Division of Medical Oncology, Department of Medicine, Faculty of Medicine Ramathibodi Hospital, Mahidol University, Bangkok, Thailand; 2 Department of Medicine, Faculty of Medicine, King Chulalongkorn Memorial Hospital and Chulalongkorn University, Bangkok, Thailand; 3 Division of Medical Oncology, Department of Medicine, Faculty of Medicine Siriraj Hospital, Mahidol University, Bangkok, Thailand; 4 Department of Medical Oncology, Chulabhorn Hospital, Chulabhorn Royal Academy, Bangkok, Thailand; 5 Division of Medical Oncology, Department of Internal Medicine, Faculty of Medicine, Prince of Songkla University, Songkhla, Thailand; 6 Medical Oncology Unit, Department of Medicine, Sawanpracharak Hospital, Nakhonsawan, Thailand

**Keywords:** Breast cancer, Palbociclib, Ribociclib, Propensity score matching

## Abstract

**Background:**

The current standard first-line treatment for hormone receptor-positive/human epidermal growth factor receptor 2 negative (HR + /HER2 −) advanced breast cancer (ABC) is a combination of aromatase inhibitor (AI) plus CDK4/6 inhibitors (CDK4/6i). Direct comparison trials of different CDK4/6i are scarce. This real-world study compared the effectiveness of first-line AI plus ribociclib versus palbociclib.

**Methods:**

This multicenter retrospective cohort study, conducted in six cancer centers in Thailand, enrolled patients with HR + /HER2 − ABC treated with first-line AI, and either ribociclib or palbociclib. Propensity score matching (PSM) was performed. The primary endpoint was overall survival (OS). Secondary endpoints included progression-free survival (PFS), overall response rate (ORR), time to chemotherapy (TTC), and adverse events.

**Results:**

Of the 250 patients enrolled, 134 patients with ribociclib and 49 patients with palbociclib were captured after PSM. Baseline characteristics were well-balanced between groups. Median PFS in patients receiving ribociclib and palbociclib were 27.9 and 31.8 months, respectively (hazard ratio: 0.87; 0.55–1.37). The median OS in the AI + ribociclib arm was 48.7 months compared to 59.1 months in the AI + palbociclib arm (hazard ratio: 0.55; 0.29–1.05). The median TTC in the AI + palbociclib group was 56 months, but not reached in the AI + ribociclib group (*p* = 0.42). The ORR of AI + ribociclib and AI + palbociclib were comparable (40.5% vs. 53.6%, *p* = 0.29). Patients receiving palbociclib demonstrated a higher proportion of neutropenia compared to those receiving ribociclib, despite a similar dose reduction rate (*p* = 0.28). Hepatitis rate was similar between the ribociclib (21%) and palbociclib groups (22%). Additionally, a low incidence of QT prolongation was observed in both the ribociclib (5%) and palbociclib groups (4%).

**Conclusion:**

This preliminary analysis of a real-world study demonstrated the comparable effectiveness of ribociclib and palbociclib with AI as an initial therapy for HR + /HER2 − ABC. No statistically significant difference in PFS, OS, and TTC was found in patients treated with AI combined with palbociclib or ribociclib. Longer follow-up and further prospective randomized head-to-head studies are warranted.

**Supplementary Information:**

The online version contains supplementary material available at 10.1186/s12885-024-12765-x.

## Background

Breast cancer is the most prevalent cancer among females globally, including Thailand [1]. Its diverse subtypes hinge on staining for hormone receptor (HR) and human epidermal growth factor receptor 2 (HER2), with HR-positive and HER2-negative (HR + /HER2 −) breast cancer being the most prevalent [2]. Controlling disease, bolstering overall survival (OS), and improving the quality of life (QoL) are the primary aims of advanced breast cancer (ABC) treatment. Developing the treatment for locally advanced/metastatic breast cancer (LA/MBC) relies on factors, including tumor subtype, disease burden, Eastern Cooperative Oncology Group performance status(ECOG-PS), comorbidities, and economic considerations [3].

The initial treatment typically is upfront hormonal therapy for patients with HR + /HER2 − LA/MBC experiencing non-visceral crises. Selective estrogen receptor modulators, non-steroidal aromatase inhibitors (NSAIs), steroidal aromatase inhibitors (SAIs), and selective estrogen receptor downregulators are among the variety of options available [2]. Recent studies indicate that initiating treatment with upfront hormonal therapy improves progression-free survival (PFS) and enhances patients’ QoL. First-line tamoxifen provided a PFS of approximately 8 months [4], whereas PFS from NSAIs and fulvestrant were approximately 12 months [4, 5] and 14 months, respectively [4].

Cyclin-dependent kinase (CDK) is a crucial molecule for cancer cell division. The interaction of cyclin D1 with CDK4 and CDK6 in the cell cycle causes hyperphosphorylation of the retinoblastoma gene (*Rb*), thereby activating the cell to pass from the G-phase checkpoint to the S phase (replication phase) of the cell cycle. Cyclin D-CDK4/6-Rb pathway alterations, such as cyclin D amplification, *Rb* gene loss or mutation, or P16 loss, cause uncontrolled cell cycle progression. Consequently, cancer cells divide rapidly and metastasize [6].

Currently, drugs that inhibit the CDK4/6-Rb pathway (CDK4/6 inhibitors [CDK4/6i]), such as palbociclib, ribociclib, and abemaciclib, have been approved as an effective therapy for ER + /HER2 − ABC. Studies have revealed the benefit of CDK4/6i when combined with NSAI as first-line treatment which prolongs PFS and increases overall response rates (ORR) compared to AI monotherapy. Data from current studies indicate the median PFS for palbociclib, ribociclib, and abemaciclib of 28, 25, and 28 months, respectively [7–9]. The ORR from the combination CDK4/6i and NSAI stands at 53%–59% compared to AI which typically yields an ORR of approximately 30%–40%. Regarding OS, palbociclib, ribociclib, and abemaciclib have reported median OS of 53.9, 63.9, and 66.8 months [7, 8, 10–13], respectively. Notably, ribociclib is the only CDK4/6i that exhibited a significant OS improvement when compared to AI monotherapy in the first-line setting. Additionally, these agents in combination with fulvestrant provide gains in PFS and ORR in the second-line setting [14–16].

The side effects of CDK4/6i vary according to the specific drug. The main side effects of palbociclib include neutropenia, whereas ribociclib includes neutropenia, QT prolongation, and hepatotoxicity. Abemaciclib is more associated with diarrhea with less frequent myelosuppression [7–9].

Presently, prospective studies reported no evidence, and real-world evidence (RWE) that directly compares the efficacy and toxicities between CDK4/6i types when combined with AI for treating patients with HR + /HER2 − LA/MBC remains limited.

Palbociclib, ribociclib, and abemaciclib obtained Thai FDA approval in 2017, 2018, and 2020, respectively. However, reported outcomes concerning their efficacy in the first-line setting, as well as comparative efficacy among the different CDK4/6i, remain lacking. Furthermore, we aim to investigate the toxicity profile of these CDK4/6i in Thai patients which may diverge from that observed in reports from Western countries.

This multicenter study aimed to investigate the efficacy differences between ribociclib and palbociclib when combined with NSAI for first-line therapy of HR + /HER2 − LA/MBC in real-life clinical practice.

## Patients and methods

### Patients

Inclusion criteria were age of ≥ 18 years and histologically or cytologically confirmed HR + /HER2 − ABC diagnosis, defined as tumors expressing estrogen and/or progesterone receptors of > 1% and HER2 negativity determined by immunohistochemistry scores of 0, 1 + , or 2 + with negative results by in situ hybridization. Additionally, patients must have received first-line treatment with AI combined with ribociclib or palbociclib. This study included menopausal or premenopausal patients receiving ovarian function suppression. All patients included in the study were diagnosed with LA/MBC from January 1, 2017, to October 31, 2022, with the last follow-up cut-off date on September 30, 2023. Exclusion criteria were insufficient or missing data and previous chemotherapy for treatment in a metastatic setting.

### Study design

This multicenter retrospective cohort study was conducted across six medical institutions/centers in Thailand. The study aimed to compare the efficacy of ribociclib and palbociclib when combined with AI as a first-line therapy for HR + /HER2 − ABC. The primary endpoint was OS by propensity score-match (PSM) analysis, whereas secondary endpoints were PFS, subgroup analysis of OS, time to chemotherapy (TTC), response rate, CDK4/6i dose modification rate, and toxicity. The Institutional Review Board of all participating institutions approved the study.

### Statistical analysis

The chi-square test or Fisher’s test was used to compare qualitative variables, whereas the Student’s *t*-test was used to compare quantitative variables. The Mann–Whitney U test was utilized to compare medians. PSM was conducted to balance baseline characteristics. The Kaplan–Meier method was used to estimate PFS, OS, and TTC with group comparisons conducted using the log-rank test. A Cox proportional hazard model was used to estimate hazard ratios.

PSM analysis was conducted to minimize potential selection bias due to the lack of randomization. Propensity scores for AI with palbociclib vs. AI with ribociclib were estimated using a logistic regression model based on clinically selected covariates, including age, ECOG-PS, de novo metastasis, visceral metastasis, and level of estrogen receptor (ER) expression (< 50% or ≥ 50%). Propensity score-adjusted analyses were conducted in the sensitivity analysis. The results are presented as hazard ratios and 95% confidence intervals (CI). The *p*-value of < 0.05 was considered statistically significant. All analyses were conducted using STATA version 14.

## Results

### Patients’ clinical characteristics

**Fig. 1:**
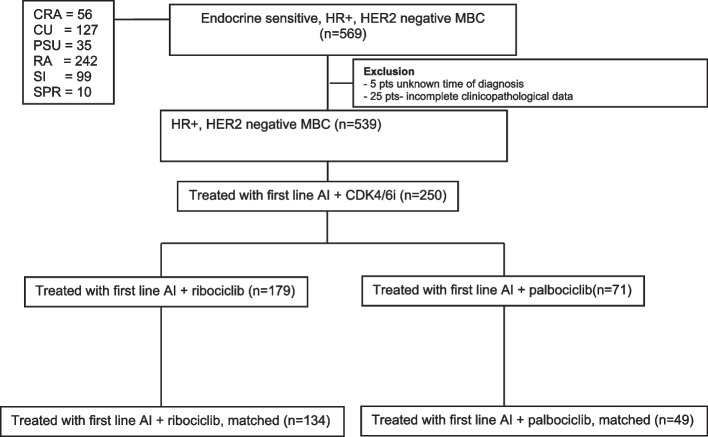
Patient consort diagram. Abbreviations: CRA: Chulabhorn Royal Academy, CU: Chulalongkorn Memorial Hospital, PSU: Prince of Songkhla University, RA: Ramathibodi Hospital, SPR: Sawanpracharak Hospital, SI: Siriraj Hospital, AI: aromatase inhibitor, HR: hormone receptor, HER-2: Human Epidermal Growth Factor Receptor 2, MBC: metastatic breast cancer, CDK4/6i: cyclin-dependent kinases 4 and 6 inhibitor

Figure 1 illustrates the patient consort diagram, indicating that an initial 539 patients with HR + /HER2 − ABC receiving first-line therapy were collected from six tertiary care centers. Of these patients, 250 received first-line treatment with a combination of AI and CDK4/6i. Out of these 250 patients, PSM analysis revealed 183 matched patients for analysis, including 134 patients receiving AI combined with ribociclib and 49 patients treated with AI combined with palbociclib.

Baseline characteristics were well-balanced between the two groups (Table 1). The median age in the ribociclib and palbociclib groups was 58 and 56 years, respectively. The majority of patients demonstrated an ECOG-PS score of 0–1 (84% in the ribociclib group vs. 87% in the palbociclib group). The proportion of patients with high ER expression (≥ 50%) was comparable in both groups (93% vs. 86%). Visceral metastases were reported in 55% and 53% of patients receiving ribociclib and palbociclib, respectively. Additionally, the majority of patients exhibited fewer than three metastatic sites (78% vs. 75%) (Table 1).

**Table 1:** Baseline characteristics

Baseline characteristics	Unmatched n(%)		Std. Difference	Matched n(%)		Std. Difference
	Ribociclib + AI (*n* = 179)	Palbociclib + AI (*n* = 71)		Ribociclib + AI (*n* = 134)	Palbociclib + AI (*n* = 49)	
**Median age (yrs.) (range)**	54 (36–72)	52(32–72)	0.11575	58(44–72)	56(37–75)	0.0216
**Menopause status**						
Postmenopausal status	130(73)	59(83)	-0.2531	109(81)	42(86)	-0.1173
Premenopausal status	49(27)	12(17)	0.22631	25(18)	7(14)	0.0539
**ECOG PS**						
- 0	51(28)	25(31)	-0.1439	32(24)	15(30)	-0.1505
- 1	102(57)	36(51)	0.12558	81(60)	28(57)	0.0667
- 2	26(15)	9(13)	0.0537	21(16)	6(12)	0.0983
- ≥ 3	0	1(1)		0	0	
**Comorbidities**						
- Diabetes	36(20)	9(13)	0.20095	28(21)	7(14)	0.1731
- Hypertension	47(26)	22(31)	-0.1042	39(29)	16(33)	-0.0763
- Dyslipidemia	33(18)	12(17)	0.04004	24(18)	7(14)	0.0981
- CAD	1(1)	0	0.1063	1(1)	0	0.1231
**Aromatase inhibitors**						
- Letrozole	175(98)	71(100)	-0.0963	130(97)	49(100)	-0.1005
- Anastrozole	4(2)	0	0.21321	4(3)	0	0.2471
**Hormone receptor status**						
- ER positive	179(100)	71(100)	0.24437	134(100)	49(100)	0.2085
- Median ER level	90	90	0.14121	86.16	82.68	0.1797
◦ ER < 50	17(9)	11(15)	-0.1468	10(7)	7(14)	-0.1629
◦ ER ≥ 50	162(91)	60(85)	0.14679	124(93)	42(86)	0.1629
- PR positive	140(78)	54(76)	0.0511	102(76)	35(71)	0.1060
**De novo metastasis**	85(47)	40(56)	-0.0638	68(51)	27(55)	0.0460
**Recurrent disease**						
- DFI < 12 mo	52(29)	17(24)	0.00955	33(25)	11(22)	0.0000
- DFI 12–24 mo	7(4)	1(1)	0.18698	6(4)	1(2)	0.1787
- DFI > 24 mo	35(20)	13(18)	-0.0952	27(20)	10(20)	-0.0904
**Previous therapy for EBC**						
Neo/adjuvant chemotherapy	74(41)	25(35)	0.19506	48(36)	17(35)	0.1046
Neo/adjuvant ET	94(53)	34(48)	0.12462	66(49)	24(49)	0.0920
Tamoxifen alone	84(47)	30(42)	0.03535	59(44)	22(45)	-0.0766
Sequential Tamoxifen/AI	10(6)	4(6)		7(5)	2(4)	
**Metastasis site**						
- Visceral (lung, liver, brain)	103(58)	34(48)	0.19332	74(55)	26(53)	0.0431
- Bone only	51(28)	25(35)	-0.1439	41(31)	15(31)	-0.0003
- Lymph node only	7(4)	3(4)	-0.0159	5(4)	1(2)	0.1005
**No. of metastasis site**						
- 1	73(41)	39(55)	-0.2847	52(39)	25(51)	-0.2457
- 2	64(37)	15(21)	0.32708	52(39)	12(24)	0.3095
- ≥ 3	42(23)	17(24)	-0.0921	30(22)	12(24)	-0.1222

*Abbreviations: AI* Aromatase inhibitor, *yrs* years, *ECOG PS* Eastern Cooperative Oncology Group performance status, *CAD* Coronary artery disease, *ER* Estrogen receptor, *PR* Progesterone receptor, *DFI* Disease free interval (defined from time of completion of adjuvant endocrine therapy to first relapse), *ET* Endocrine therapy, *EBC* Early breast cancer

### Comparative effectiveness analysis of palbociclib and ribociclib

#### Overall survival

The data cut-off date for OS analysis was September 30, 2023. The median follow-up time was 29 months (95% CI: 26.15–31.85). Death events occurred in 45 (33%) of 134 and 15 (30%) of 49 patients in the ribociclib and palbociclib groups, respectively.

**Fig. 2:**
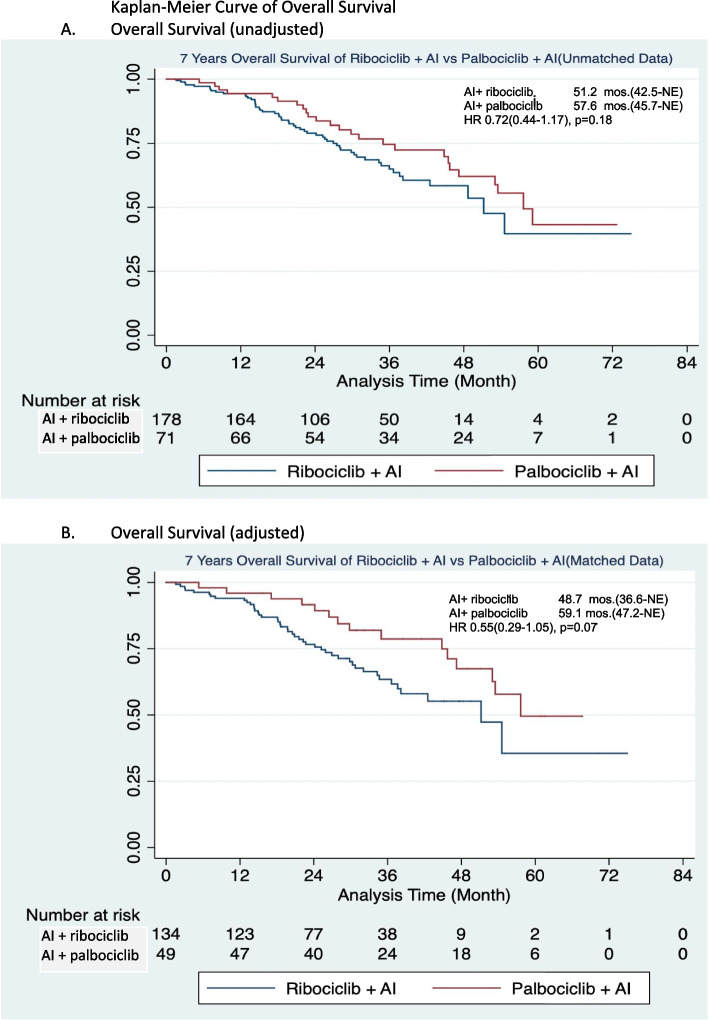
Kaplan–Meier curve of overall survival. **A** Overall Survival (unadjusted). **B** Overall Survival (adjusted). Abbreviations: AI: aromatase inhibitor, mos: months, HR: hazard ratio, NE: not estimate

The unadjusted analysis of the entire cohort revealed the comparable median survival between the ribociclib and palbociclib groups at 51.2 months and 57.6 months, respectively (hazard ratio: 0.72, 95% CI: 0.44–1.17, *p* = 0.18) (Fig. 2A). Following PSM analysis, the adjusted median OS demonstrated a trend toward shorter OS with ribociclib + AI (48.7 months among patients receiving ribociclib and 59.1 months in the palbociclib group (hazard ratio of death: 0.55, 95% CI: 0.29–1.05, *p*-value: 0.07) (Fig. 2B).

**Fig. 3:**
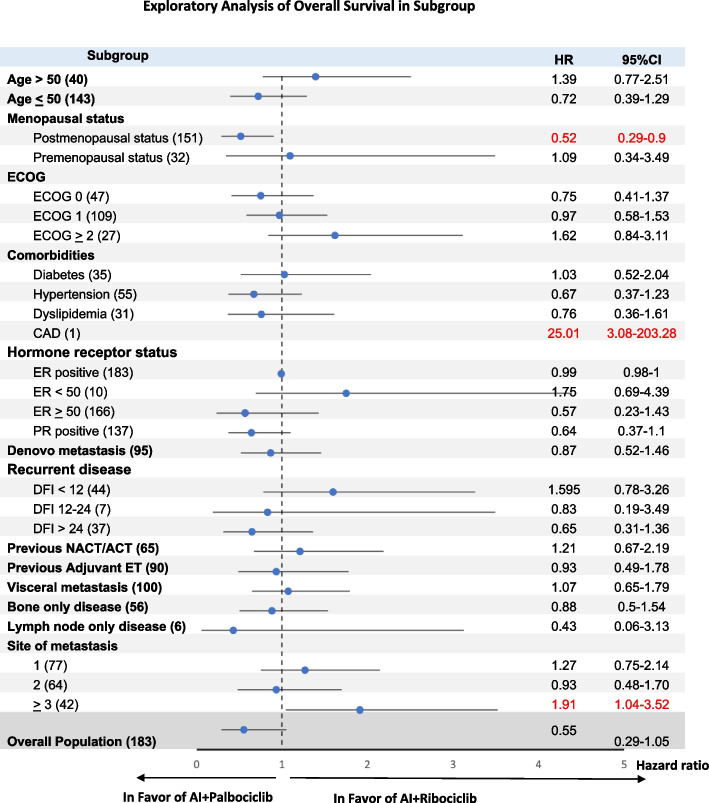
Exploratory analysis of overall survival in subgroup. Abbreviations: AI: aromatase inhibitor, yrs: years, ECOG PS: Eastern Cooperative Oncology Group performance status, CAD: coronary artery disease, ER: estrogen receptor, PR: progesterone receptor, DFI: disease-free interval, ET endocrine therapy, HR: hazard ratio, CI: confidence interval

A subgroup analysis of OS revealed no preferential benefit of either palbociclib or ribociclib (Fig. 3) across baseline characteristics of the patients. OS benefit was observed with ribociclib among patients with ≥ 3 metastasis sites and patients with coronary disease. However, the 95% CI was very wide and should be interpreted with caution.

#### Progression free survival

**Fig. 4:**
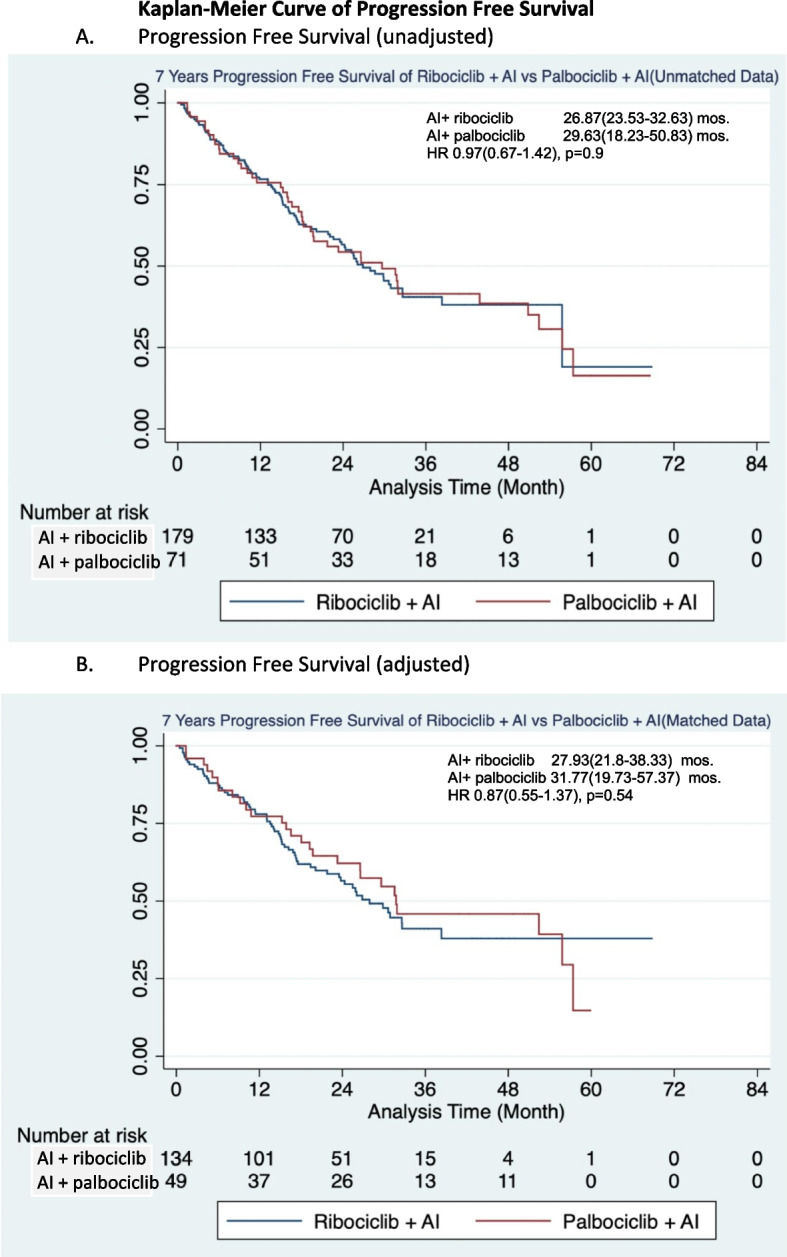
Kaplan–Meier curve of progression free survival. **A** Progression free survival (unadjusted). **B** Progression free survival (adjusted). Abbreviations: AI: aromatase inhibitor, mos: months, HR: hazard ratio, NE: not estimate

Of 250 patients with HR + /HER2 − ABC receiving first-line AI + CDK4/6i, the median PFS was 26.9 (95% CI: 23.5–32.6) and 29.6 (95%CI 18.2–50.8) months (hazard ratio: 0.97, 95% CI: 0.7–1.4) in the ribociclib and palbociclib groups, respectively. A consistent indifference in the median PFS was observed from 180 patients available for analysis after PSM, including 27.9 months (95% CI: 21.8–38.3) and 31.8 months (95% CI: 19.7–57.4) in the ribociclib and palbociclib groups, respectively (hazard ratio: 0.87, 95%CI 0.55–1.37) (Fig. 4).

#### Univariate and multivariate analysis of overall survival

A univariate analysis identified age at diagnosis, ECOG-PS, menopausal status, comorbidities, de novo metastasis, and the number of metastatic sites to be associated with OS. Only four factors remained independently associated with OS after multivariable analysis (Table 2). The favorable prognostic factors include having one metastatic site and postmenopausal status, whereas adverse prognostic factors include worsening of ECOG-PS and the presence of coronary artery disease. Notably, different CDK4/6i types (ribociclib vs. palbociclib) were not associated with OS outcome in both univariate and multivariate analyses.

**Table 2:** Univariate and multivariate analysis for overall survival

	Univariate analysis			Multivariate analysis		
	HR	*P*- Value	95%CI	HR	*P-* Value	95%CI
**AI + ribociclib**	Reference			Reference		
**AI + palbociclib**	0.55	0.07	0.29–1.05	0.51	0.06	0.28–1.04
**Age (per 1 year)**	1.02	0.00	1.01–1.03	1.00	0.61	0.99–1.02
**ECOG status (per 1 ECOG)**	1.51	< 0.05	1.20–1.89	3.01	0.03	1.08–8.37
**Menopausal status**						
Pre-menopausal	Reference			Reference		
Post-menopausal	0.63	0.02	0.43–0.92	0.16	0.01	0.04–0.62
**Co-morbidites**						
Diabetes	1.29	0.14	0.92–1.82			
Hypertenstion	0.92	0.55	0.62–1.22			
Dyslipidemia	0.87	0.43	0.60–1.24			
CAD	3.16	0.01	1.40–7.13	2.83	0.02	1.16–6.88
**Hormonal receptor status**						
ER < 50	Reference					
ER ≥ 50	0.74	0.09	0.52–1.04			
ER positive (per 1%)	1.32	0.70	0.33–5.30			
PR positive (per 1%)	0.90	0.49	0.67–1.21			
**Denovo metastasis**	1.29	0.05	1.00–1.67	1.18	0.34	0.84–1.66
**Recurrent disease**						
DFI < 12	Reference					
DFI 12–24	0.84	0.61	0.42–1.66			
DFI > 24	0.92	0.67	0.63–1.34			
**Previous NACT/ACT**	0.86	0.29	0.64–1.14			
**Previous Adjuvant ET**	0.87	0.35	0.65–1.16			
**Visceral metastasis**	1.16	0.25	0.90–1.50			
**Bone only disease**	0.93	0.59	0.71–1.21			
**Lymph node only disease**	0.70	0.39	0.31–1.51			
**No. of metastasis site**						
1 site	Reference			Reference		
2 sites	1.24	0.12	0.94–1.63			
≥ 3 sites	1.597	0.0040	1.16–2.20	1.23	0.29	0.84–1.80

*Abbreviations: AI* Aromatase inhibitor, *ECOG PS* Eastern Cooperative Oncology Group performance status, *CAD* Coronary artery disease, *ER* Estrogen receptor, *PR* Progesterone receptor, *DFI* Disease free interval (defined from time of completion of adjuvant endocrine therapy to first relapse), *ET* Endocrine therapy, *EBC* Early breast cancer, *HR* Hazard ratio, *CI* Confident interval

#### Time to chemotherapy and total lines of treatment

**Fig. 5:**
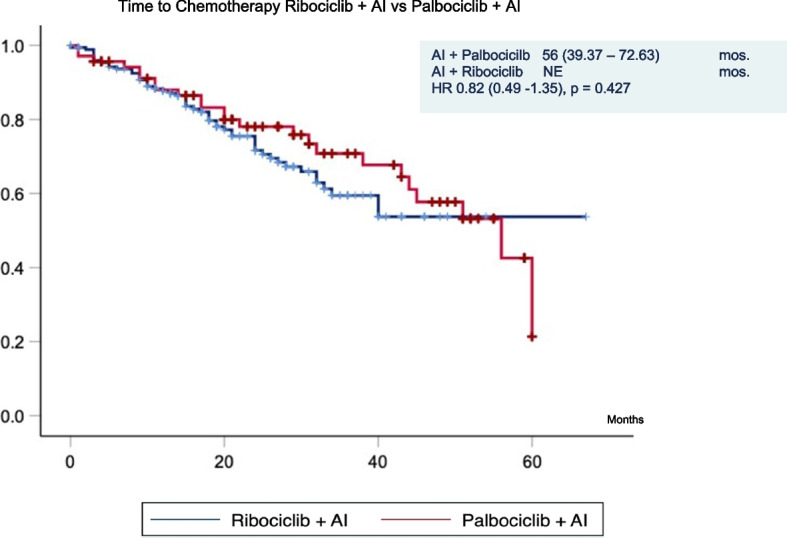
Time to chemotherapy.  Abbreviations: AI: aromatase inhibitor, mos: months, HR: hazard ratio, NE: not estimate

Time to first chemotherapy was comparable between both groups (Fig. 5). The median TTC was 56 months (95% CI: 39.37–72.63) in the palbociclib group while in the ribociclib group it was not reached (*p* = 0.42). Patients who received palbociclib had a trend to receive more subsequent therapy with a median of three lines (95% CI: 2.05–4.08) compared to two (95% CI: 1.8–2.8) lines in the ribociclib group (*p* = 0.3).

#### Response rate

The ORRs in the AI + ribociclib and AI + palbociclib groups were 40.5% and 53.6%, respectively (*p* = 0.29). Similarly, the disease control rate was excellent in both groups (89.6% in the ribociclib group and 92.7% in the palbociclib group, *p* = 0.29). The median time to response was 15.5 (95% CI: 13.57–17.71) and 11.7 (95% CI: 11–37.14) weeks among patients receiving AI + ribociclib and AI + palbociclib (*p* = 0.12), respectively.

#### Toxicity

Neutropenia and anemia are common toxicities observed in both groups. Grades 3–4 neutropenia was significantly less frequent among patients with ribociclib therapy (48% vs. 69%, *p* = 0.02). Additionally, grades 3–4 thrombocytopenia occurred in only 2% of patients in the ribociclib group compared to 8% in the palbociclib group (*p* = 0.001). Grades 3–4 anemia was numerically more frequently observed in patients receiving palbociclib (6% vs. 3%, *p* = 0.44). Abnormal aspartate transaminase/alanine aminotransferase elevation, mostly in grades 1–2, was seen in 19% and 22% of patients in the ribociclib and palbociclib groups, respectively (*p* = 0.59). QTc prolongation was reported in 5.1% and 4% of patients in the ribociclib and palbociclib groups, respectively (*p* = 0.91) (Table 3).

**Table 3:** Side effects

Side effects	AI + ribociclib (*n* = 134)				AI + palbociclib (*n* = 49)				*P*—Value
Grade	1	2	3	4	1	2	3	4	
**Anemia**	32(24%)	17(13%)	4(3%)	0	12(26%)	8(16%)	3(6%)	0	0.444
**Neutropenia**	12(9%)	34(25%)	60(45%)	4(3%)	3(6%)	4(9%)	30(61%)	4(8%)	0.022
**Thrombocytopenia**	9(7%)	4(3%)	3(2%)	0	9(19%)	3(6%)	4(8%)	0	0.001
**Hepatitis (AST or ALT)**	17(13%)	8(6%)	3(2%)	0	8(17%)	2(5%)	0	0	0.594
**Nausea**	5(4%)	5(4%)	3(2%)	0	0	0	0	0	0.144
**QTc prolongation**	2(1.5%)	1(0.7%)	4(2.9%)	0	1(2%)	0	1(2%)	0	0.911

*Abbreviations: AI* Aromatase inhibitor, *AST* Aspartate aminotransferase, *ALT* Alanine aminotransferase

### Real-world practice of CDK4/6i dosing and dose modification

Nearly all patients (98.6%) in the palbociclib group received the full starting dose of 125 mg daily whereas 86.6% in the ribociclib group received the starting dose of 600 mg daily (*p* = 0.004). A higher proportion of patients receiving palbociclib experienced myelosuppression, but the rate of CDK4/6i dose reduction was similar (65% and 60% among ribociclib and palbociclib, respectively). All dose reductions occurred in patients who were started on full doses of both agents except for one patient receiving ribociclib at a starting dose of 400 mg who still required a dose adjustment. A similar proportion of patients in both groups required a one-dose level reduction (49% vs. 48%). Additionally, 16% and 11% of patients in the ribociclib and palbociclib groups, respectively, underwent a two-dose level reduction. The median time to dose reduction was 42 days (95% CI: 27.35–56.65) and 49 days (95% CI: 14.31–83.69) in the ribociclib and palbociclib groups, respectively (*p* = 0.01).

## Discussion

CDK4/6i in combination with AI has revolutionized the treatment of HR + /HER2 − ABC and become a standard of care globally. An unsurpassed PFS gain over that of ET alone has been consistently reported in pivotal randomized controlled trials (RCTs) of palbociclib [7], ribociclib [8, 17], and abemaciclib [9] whereas the OS improvement was statistically and/or clinically significant with ribociclib [8, 10] and abemaciclib [13], respectively. However, no RCT and only a few RWEs directly compared the OS of ribociclib and palbociclib [18, 19].

Our study investigated the outcome of first-line treatment with palbociclib or ribociclib in combination with AI for HR + /HER2 − ABC. We collected real-world practice data from six centers across Thailand. A PSM was used to balance the patients’ clinicopathological characteristics and minimize the bias of the retrospective nature of real-world data. We also included ER expression levels using the cut-off level of 50% in both the baseline characteristics and efficacy analysis. The 50% cutoff was chosen as a surrogate for endocrine responsiveness which has been demonstrated in various settings [20–23]. We revealed no statistically significant differences in both OS and PFS between palbociclib and ribociclib as first-line treatment in our cohort. A multivariate Cox regression analysis, showing a hazard ratio for the survival of 0.51 (*p* = 0.06, 95% CI: 0.8–1.04) confirmed this result.

The decision to select one agent over the other remained upon physicians’ judgment/experiences together with the side effect profiles and the patients themselves due to the lack of direct RCT comparing the effectiveness of the currently available CDK4/6i. Thus, a comparative analysis of the RWE was used to decipher the dilemma and contribute complementary information to that of RCT. As abemaciclib was the last agent in this class to receive approval in Thailand, we only compared the efficacy of palbociclib and ribociclib in a real-world situation with the main interest in OS. Both the adjusted OS and PFS of our palbociclib cohort were not statistically different from that of ribociclib. These results were congruent with other RWE reports that compared ribociclib and palbociclib outcomes [18, 19]. Numerically, the OS and PFS of both groups were consistent with those of large pivotal trials (PALOMA-2, MONALEESA-2, and MONALEESA-7), except for a somewhat lower OS in our ribociclib cohort (48.7 months vs. 63.9 and 58.7 months in MONALEESA-2 and MONALEESA-7, respectively) [8, 10]. Notably, compared to other RWE of individual CDK4/6i, our study demonstrated longer PFS in both treatment arms [7, 17, 24–28]. Several factors may have contributed to these differences. Patients in our cohort exhibited a lower tumor burden, with > 80% having fewer than three metastatic sites and > 90% having high ER expression (ER ≥ 50%) compared to other trials, thereby representing an endocrine-sensitive population. These results demonstrate that palbociclib and ribociclib are highly effective in endocrine-sensitive patients with low tumor burden and high ER expression. Several factors may have contributed to the numerically shorter OS in ribociclib arm in our study. We revealed a higher number of subsequent therapies in the palbociclib group which could affect OS. In addition, as palbociclib was the first CDK4/6i approved in Thailand, which resulted in longer follow-up periods and potentially more accurate OS assessment in this group.

The results of our study were in contrast to an analysis by Jhaveri et al. [29], which uses a matching adjusted indirect comparison to individual data from MONALEESA-2 and PALOMA-2, demonstrating a greater OS in favor of ribociclib as a first-line regimen. However, its generalizability to a broader patient population in practice is limited, considering the strict and narrow inclusion/exclusion in RCT.

The ORR of the treatment was comparable between the two groups (48% with ribociclib vs. 53.6% with palbociclib). Our real-world finding aligned with MONALEESA and PALOMA-2 trials [7, 12, 17] and previous RWE trials [30–34].

Our study emphasized neutropenia as the primary adverse event as well as a higher myelosuppression incidence with palbociclib compared to ribociclib in terms of tolerability. This indicated a greater bone marrow toxicity in palbociclib compared to ribociclib, possibly due to pharmacokinetic variances [35, 36]. Nearly all patients in the palbociclib group received a full starting dose compared to only 86.6% in the ribociclib group. A similar rate of dose reduction was demonstrated, mostly due to myelosuppression, and this rate was congruent with other reports from Asian [25, 37] and Western populations alike [28, 38].

This study represents the first and largest multicenter cohort data in the real-world practice of first-line ribociclib + AI versus palbociclib + AI in metastatic HR + , HER- breast cancer in Southeast Asia, thereby providing valuable insights into the context of a non-Western population. Importantly, the absence of randomized phase 3 trials comparing the efficacy between palbociclib + AI and ribociclib + AI emphasizes the significance of this study in addressing this literature gap. Despite its strengths, this study contained limitations. Firstly, being retrospective in nature, inherent bias may exist in patient selection at the outset. However, we mitigated this bias by using a PSM. Secondly, our study lacked data on patient-reported outcomes, QoL, and economic outcomes. Thirdly, information on subsequent treatment regimens was incomplete. In addition, this study involves a relatively small number of patients and also with imbalance of numbers of patients in palbociclib arm as only ribociclib was reimbursed in Thailand. And lastly, the median follow-up time of the cohort is relatively short compared to others as the drugs have just received approval in Thailand in 2017. Thus, longer follow-up and update of the current result would be vital to confirm the real effectiveness of both agents.

In summary, our RWE from Thai population indicated no differences in overall outcomes, TTC, and response rates between ribociclib and palbociclib as a first-line therapy despite the inconsistent OS gain of first-line ribociclib and palbociclib in RCT.

### Supplementary Information

Supplementary Material 1. Supplementary Appendix Fig. 1. Distribution of the propensity score

## Data Availability

The datasets used and/or analyzed during the current study are available from the corresponding author on reasonable request.

## Abbreviations

ABC: Advanced breast cancer
CDK: Cyclin-dependent kinase
ECOG: Eastern Cooperative Oncology Group
ER: Estrogen receptor
ET: Endocrine therapy
HR: Hormone receptor
HER2: Human epidermal growth factor receptor 2
LA/MBC: Locally advanced/metastatic breast cancer
NSAIs: Non-steroidal aromatase inhibitors
ORR: Overall response rate
OS: Overall survival
PFS: Progression free survival
PR: Progesterone receptor
PS: Performance status
PSM: Propensity score matching
QoL: Quality of life
Rb: Retinoblastoma gene
RCT: Randomized controlled trial
RWE: Real-world evidence
SAIs: Steroidal aromatase inhibitors
TTC: Time to chemotherapy
