# Supplementary material for: Comparative effectiveness analysis of survival with first-line palbociclib or ribociclib plus AI in HR + /HER2- advanced breast cancer (CEPRA study): preliminary analysis of real-world data from Thailand
Source: BMC Cancer. 2024 Aug 16;24:1018. doi: 10.1186/s12885-024-12765-x (PMC11328387; doi:10.1186/s12885-024-12765-x)
Supplement: Supplementary file 1 — Supplementary Material 1. Supplementary Appendix Fig. 1. Distribution of the propensity score [file 12885_2024_12765_MOESM1_ESM.pdf]

Supplementary Appendix

Figure 1. Distribution of the Propensity Score

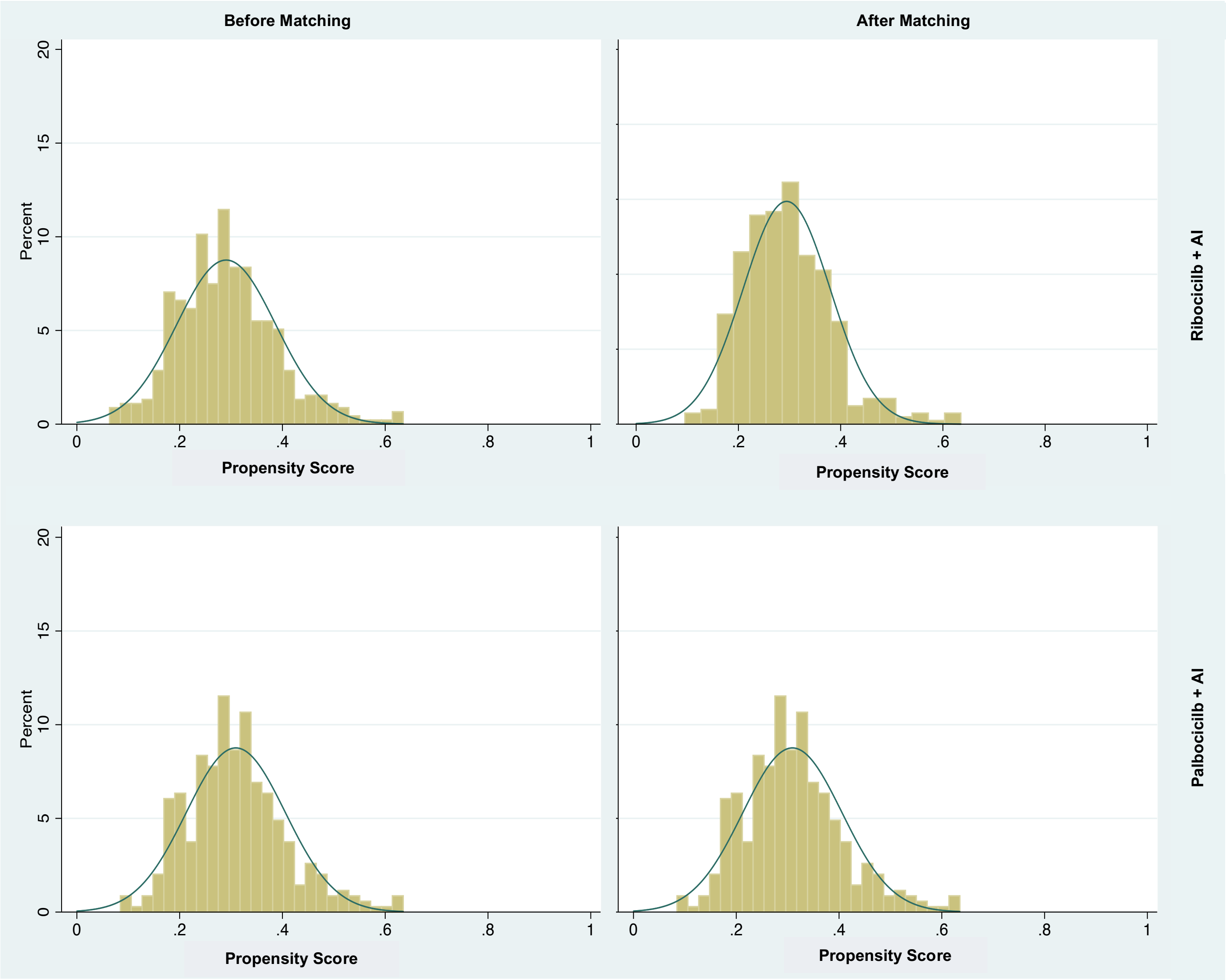

Abbreviations: AI: aromatase inhibitor
